# RA Fibroblast-Like Synoviocytes Derived Extracellular Vesicles Promote Angiogenesis by miRNA-1972 Targeting p53/mTOR Signaling in Vascular Endotheliocyte

**DOI:** 10.3389/fimmu.2022.793855

**Published:** 2022-03-08

**Authors:** Yixiong Chen, Junlong Dang, Xiaorong Lin, Manli Wang, Yan Liu, Jingrong Chen, Ye Chen, Xiqing Luo, Zuoyu Hu, Weizhen Weng, Xiaoyi Shi, Xuan Bi, Yan Lu, Yunfeng Pan

**Affiliations:** ^1^Division of Rheumatology, Department of Internal Medicine, The Third Affiliated Hospital of Sun Yat-sen University, Guangzhou, China; ^2^Department of Rheumatology, Affiliated Dongguan People’s Hospital, Southern Medical University, Dongguan, China; ^3^Department of Clinical Immunology, The Third Affiliated Hospital of Sun Yat-Sen University, Guangzhou, China; ^4^Medical Research Center, The Eighth Affiliated Hospital, Sun Yat-sen University, Shenzhen, China

**Keywords:** rheumatoid arthritis, fibroblast-like synoviocytes, extracellular vesicles, miR-1972, angiogenesis

## Abstract

Rheumatoid arthritis (RA) is an autoimmune disease characterized by chronic inflammatory in joints. Invasive pannus is a characteristic pathological feature of RA. RA fibroblast-like synoviocytes (FLSs) are showed tumor-like biological characters that facilitate pannus generation. Importantly, it has been documented that extracellular vesicle (EVs) derived microRNAs have a vital role of angiogenesis in various immune inflammatory diseases. However, whether RA FLSs derived EVs can facilitate angiogenesis and the underlying mechanism is undefined. Herein, we aim to investigate the key role of RA FLSs derived EVs on angiogenesis in endothelial cells (ECs). We indicate that RA FLSs derived EVs promote ECs angiogenesis by enhancing migration and tube formation of ECs *in vitro*. Also, we confirm that RA FLSs derived EVs can significantly facilitate ECs angiogenesis with a matrigel angiogenesis mice model. In terms of the mechanisms, both RNAs and proteins in EVs play roles in promoting ECs angiogenesis, but the RNA parts are more fundamental in this process. By combining microRNA sequencing and qPCR results, miR-1972 is identified to facilitate ECs angiogenesis. The blockage of miR-1972 significantly abrogated the angiogenesis stimulative ability of RA FLSs derived EVs in ECs, while the overexpression of miR-1972 reversed the effect in ECs. Specifically, the p53 level is decreased, and the phosphorylated mTOR is upregulated in miR-1972 overexpressed ECs, indicating that miR-1972 expedites angiogenesis through p53/mTOR pathway. Collectively, RA FLSs derived EVs can promote ECs angiogenesis *via* miR-1972 targeted p53/mTOR signaling, targeting on RA FLSs derived EVs or miR-1972 provides a promising strategy for the treatment of patients with RA.

**Graphical Abstract:**
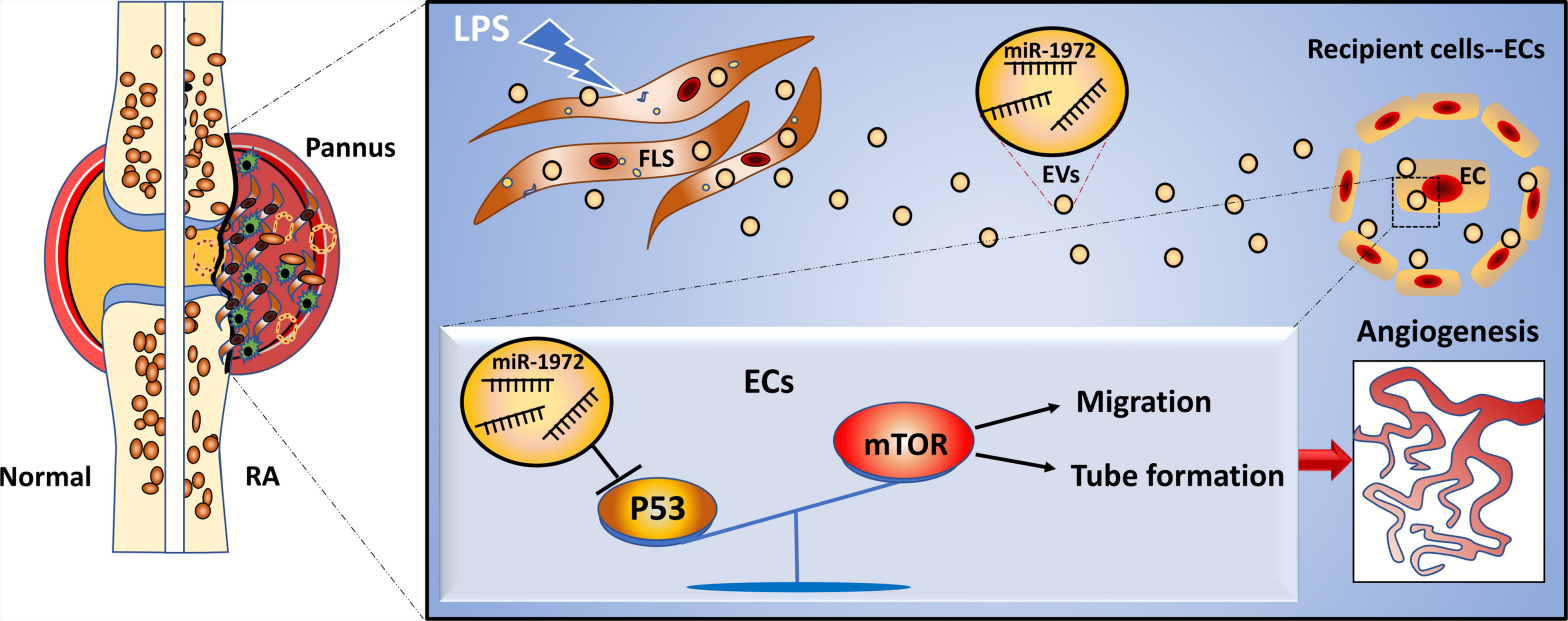


## Introduction

Rheumatoid arthritis (RA) is a systemic autoimmune disease principally effecting polyarticular synovitis and bone loss (1). In RA, synovial pannus is a driving pathological process that result in joint erosion (2). RA fibroblast-like synoviocytes (FLSs) are the predominant cell type in synovial intima (3). Furthermore, numerous evidences have indicated that pro-inflammatory cytokines activated RA FLSs possess lots of biological characteristics are similar to tumor cells, which further result in pannus generation and bone erosion *via* interacting with immune cells (4, 5). Therefore, targeting on RA-FLSs or combined with immune suppression can exert a therapeutic effect on RA (6). So far, the main pharmacologic treatments of RA are nonsteroidal anti-inflammatory drugs (NSAIDs), conventional and biologic disease-modifying antirheumatic drugs (DMARDs). However, about 30%-50% of RA patients cannot response to traditional DMARDs adequately (7). Interestingly, pirfenidone (PFD), a vascular targeted candidate, has been verified to suppress endothelial cells (ECs) migration and angiogenesis (8). Moreover, PFD can mitigate the pathological changes of CIA rats, and may serve as a potential drug for the treatment of RA (9).

Extracellular vesicles (EVs) are large parts of membrane-bound microparticles/microvesicles, apoptotic bodies, and exosomes (10). It has been accepted that EVs can mediate cell-to-cell communication by transferring their contents to target cells, alter cell transcription, and cell biological behaviors (11–13). Importantly, EVs exert functions predominantly relay on their contents, such as microRNA (miRNA), mRNA, and protein (14, 15). RA FLSs derived EVs have been documented to aggravate the severity of RA. They are shown to contain membrane-bound TNF-α, which further activate RA FLSs (16). The activated RA FLSs derived EVs can in turn exacerbate inflammatory and facilitate T cells resistant to apoptosis, forming a T cell-to-RA FLSs interaction feedback loop (16). Moreover, the active RA FLSs secrete pro-inflammatory cytokines, such as IL-6 and IL-8, which result in joint inflammation (17). Also, the RA FLSs produce matrix metalloproteinase-1 (MMP-1), MMP-3, MMP-9, and MMP-13, induce extracellular matrix (ECM) destruction and joint tissue breakdown (18). RA FLSs have been reported to activate endothelium and promote angiogenesis (19). Interestingly, CD13 was present in RA biological fluid (plasma, synovial fluid, FLSs culture supernatant), which presented in EVs (20). CD13 has been documented to promote ECs migration, tube formation *in vitro* and angiogenesis *in vivo (* 21). Besides, it also has been reported that 80% ID1 in synovial fluid was wrapped in EVs of RA FLSs, the latter can further transmit into ECs to facilitate angiogenesis (19). Thus, it is clear that protein enveloped in RA FLSs derived EVs can promote ECs angiogenesis. However, whether miRNA involved in RA FLSs derived EVs can promote angiogenesis and the underlying mechanism remain unknown.

MicroRNAs (miRNAs) are short endogenous RNAs 19 to 25 nucleotides in size that modulate post-transcriptional silencing of target genes in plants and mammals (22–24). miRNA expression in RA FLSs has been identified to lead to the production of pro-inflammatory cytokines or metalloproteinases, increased proliferation and survival in RA FLSs (25). We have previously reported that RA FLSs derived miR-221-3p facilitate the tumor-like behavior of RA FLSs, down regulation of miR-221-3p can mitigate the tumor-like character (26, 27). Simultaneously, miRNA in RA FLSs derived exosomes have the potential to ignite local inflammation and attenuate osteoclastogenesis (25). Importantly, it has been documented that miRNA in EVs have the capacity to regulate the angiogenesis in cardiovascular and cerebrovascular diseases, immune inflammatory diseases, diabetes and tumors (28–30). However, whether RA FLSs derived miRNAs can regulate the angiogenesis is unclear.

miR-1972 is a rarely investigated miRNA that modulate the process of cancer. miR-1972 has been identified to affect cell viability, invasion and metastasis *via* a ceRNA network in osteosarcoma (31, 32). Additionally, it has been documented that a APCDD1L-AS1-miR-1322/miR-1972/miR-324-3p-SIRT5 axis facilitated icotinib-resistance by suppressing autophagic degradation of EGFR in lung adenocarcinoma. Interestingly, miR-1972 has been verified to decrease the proliferation, and/or migration as well as tube formation of ECs in preeclampsia (33). However, whether miR-1972 exert the similar role in RA is unknown.

In this study, we investigated the key role of RA FLSs derived EVs on angiogenesis in vascular endotheliocyte. RA FLSs derived EVs expedited angiogenesis by enhancing endothelial cell migration and tube formation. Also, we confirmed that RA FLSs derived EVs can significantly facilitate angiogenesis of ECs with a matrigel angiogenesis mice model. We further observed both RNA and protein of EVs played roles in promoting ECs angiogenesis, but the component of RNA was more fundamental in this process. Using microRNA sequencing, miR-1972 in RA FLSs derived EVs were identified to modulate ECs angiogenesis *in vitro* and *in vivo*. The blockage of miR-1972 significantly abrogated the angiogenesis stimulative ability of RA FLSs derived EVs in ECs, while the overexpression of miR-1972 reversed the effect in ECs. Importantly, we also indicated that the p53 level was decreased, and the phosphorylated mTOR was upregulated in miR-1972 overexpressed ECs. Collectively, RA FLSs derived EVs can promote ECs angiogenesis *via* miR-1972 targeted p53/mTOR signaling, targeting on RA FLSs derived EVs or miR-1972 provides a promising strategy for the treatment of patients with RA.

## Results

### Identification and Intracellular Localization of RA FLSs Derived EVs

**Figure 1:**
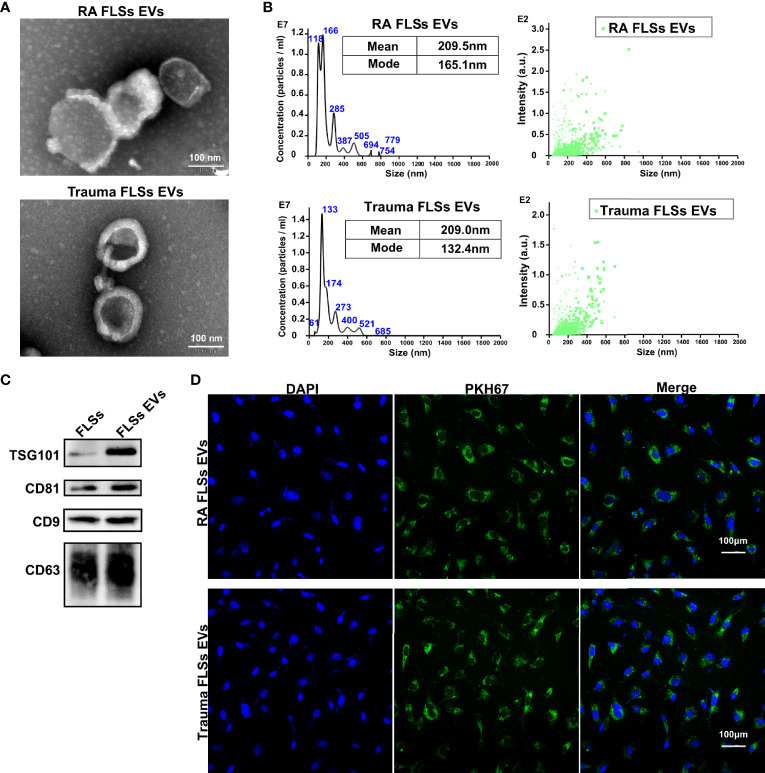
Identification and intracellular localization of RA FLSs derived EVs. RA FLSs were obtained from RA patients. Cells were maintained in DMEM and passages 3-6 were used for the follow-up experiments. EVs were prepared as the established procedure. **(A)** Representative image of EVs photographed using transmission electron microscope (TEM). **(B)** Representative results of nanoparticle tracking analyses of EVs. **(C)** EVs markers of CD9, CD63, CD81 and TSG101 were showed by Western Blot. **(D)** Immunofluorescence image to show the interaction between ECs and EVs (Nucleus: blue, EVs: green). FLSs, fibroblast-like synoviocytes; ECs, endothelial cells; EVs, extracellular vesicles.

The obtained RA FLSs or Trauma FLSs were identified by morphology imaging and flow cytometry. Both shape imaging and flow cytometry results indicated that RA FLSs and trauma FLSs shared similar morphology and phenotype (**Figure S1**). To obtain a dependable EVs, FLSs were cultured in EVs free FBS to avoid contamination of EVs from serum. Then, FLSs derived EVs were isolated from the supernatant according to the standard procedure. To be faithfully, we identified the obtained EVs from RA patients and trauma patients to confirm their purity. Using transmission electron microscopic image, we confirmed that both RA FLSs and trauma FLSs derived EVs were cup-shaped or spherical in morphology (**Figure 1A**). Moreover, we also measured the particle sizes distribution of the EVs. The results showed that the average particle sizes of the two EVs were at 209 nm (**Figure 1B**), which were consistent with the documented EVs in size (34). Next, we determined the EVs associated markers using western blotting to further confirm the EVs specificity. As expected, were showed CD9, CD63, CD81 and TSG101 were showed in the obtained EVs (**Figure 1C**). It is of vital that whether EVs can internalize into target cell and exert their functions. Therefore, we ought to assess whether EVs from RA FLSs and Trauma FLSs could be internalized by ECs. PKH67 (green) fluorescent labeled EVs were co-cultured with ECs, and the target ECs were labeled with DAPI. The immunofluorescence results indicated that both RA FLSs and Trauma FLSs derived EVs (green) were localized in the cytoplasm of ECs, and gathered around the nucleus of the target cells (blue), indicating that EVs were taken in by ECs (**Figure 1D**). Thus, by combining all those results above, we confirm the isolated EVs are dependable and have the capacity to localized in ECs.

### RA FLSs Derived EVs Promote Tube Formation in ECs

**Figure 2:**
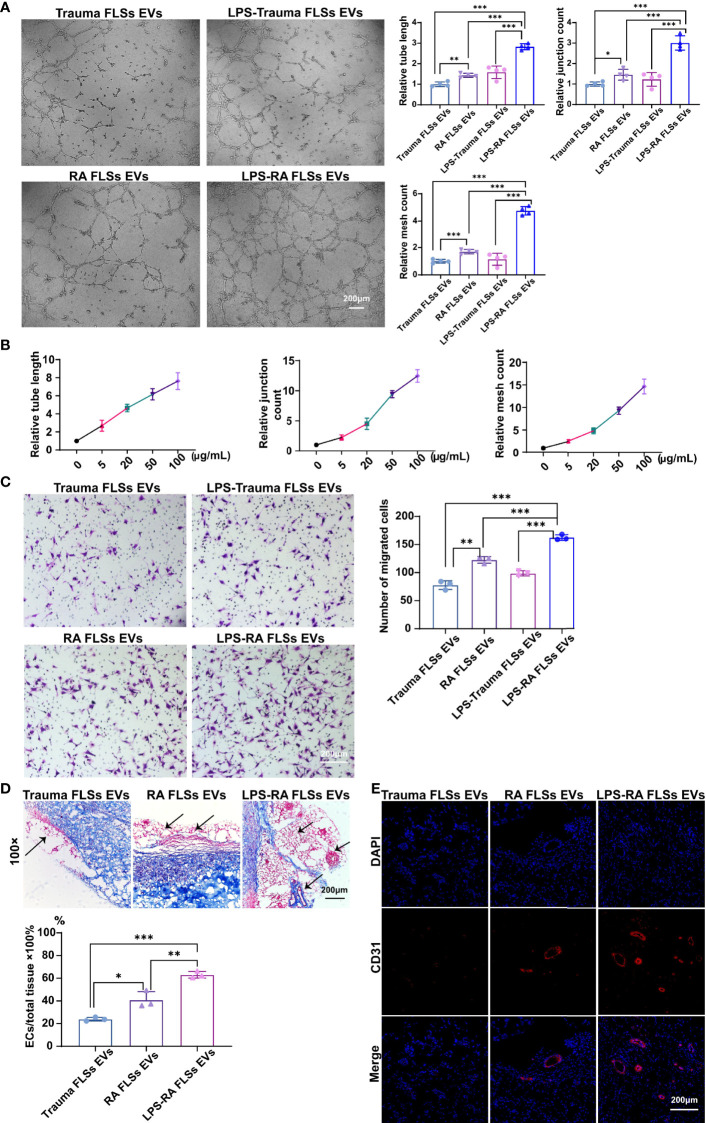
RA FLSs derived EVs promote tube formation in ECs *in vitro* and *in vivo*. **(A–C)** RA FLSs or Trauma FLSs derived EVs were obtained using the established procedure and cultured with ECs in tubule formation system or migration system. For LPS stimulation, RA FLSs were cultured in DMEM with LPS (1 μg/mL) for 3 days. **(A)** The tubule formation capacity of RA FLSs derived EVs was determined. Representative photos (50×) were obtained using microscope imaging (panel upper). The results were summarized by calculating the related parameters (panel lower). **(B)** The dose-dependent effect of RA FLSs derived EVs stimulation of ECs tubule formation *in vitro*. The relative parameters are given. The line graph describes a summary of experiments at relative parameters as indicated (n = 3). **(C)** The migration capacity of RA FLSs derived EVs was determined by transwell chamber system. The final concentration of EVs was 50 μg/mL. Typical photos (100×) (panel left), summary data (panel right) are shown. The data indicate the mean ± S.E.M of three independent experiments. **(D, E)** FLSs derived EVs expedite tubule genesis in matrigel angiogenesis mice model. Matrigel mixed with ECs and EVs was injected subcutaneously into NOD SCID mice. Two weeks post the transplantation, blood vessels were analyzed with Masson and CD31 fluorescence staining. **(D)** Masson staining results. Typical photos (100×) (panel upper), summary data (panel lower) are shown. (Red represented endothelial cells and blood vessels, blue represented matrix gel; n=3). **(E)** CD31 fluorescence staining. Typical photos (100×) are shown. FLSs, fibroblast-like synoviocytes; ECs, endothelial cells; EVs, extracellular vesicles; LPS, lipopolysaccharide. (*P < 0.05, **P < 0.01, ***P < 0.001).

Invasive pannus generation is a driving pathological process that result in joint erosion (2). The *in vitro* angiogenesis is often evaluated by the capacity of ECs to sprout, migrate, and form vascular tubules in a matrigel system (35). Therefore, we investigated the capacity of RA FLSs derived EVs to ECs angiogenesis, using transwell chamber system and matrigel system, individually. Trauma patients derived FLSs (Trauma FLSs) were used as negative control since they share similar morphological characteristics but lack pro-inflammatory properties to exclude the non-specific role of RA FLSs in angiogenesis assays. As expected, RA FLSs derived EVs dramatically facilitated tubules generation in matrigel system compared to Trauma FLSs derived EVs (**Figure 2A** , left panel). Both the relative tube length, relative junction count and relative mesh count in RA FLSs derived EVs were significantly higher than the control EVs (**Figure 2A**). Specifically, we also investigated the gradient effects of the angiogenesis of RA FLSs derived EVs at the concentration of 5 μg/mL to 100 μg/mL and the results indicated a dose-dependent effect (**Figure 2B**). Moreover, we further explored the capacity of RA FLSs derived EVs to expediting the migration in ECs. Our data showed that RA FLSs derived EVs can also facilitate the ECs migration compared to the control EVs (**Figure 2C** , left). However, RA FLSs derived EVs did not promote ECs proliferation at 24, 48 and 72 hours compared to the control EVs (**Figure S2**), indicating that RA FLSs derived EVs specifically facilitate ECs angiogenesis without affecting their proliferation at least within 24 hours. Thus, these data indicate that RA FLSs derived EVs promote angiogenesis by facilitating ECs tubule formation and migration.

The stimulative function of RA FLSs derived EVs on ECs *in vitro* does not necessarily reflect their functional capacity *in vivo*. Therefore, we developed a matrigel angiogenesis mice model. Briefly, matrigel mixed with ECs and EVs was injected subcutaneously into NOD SCID mice. Two weeks post the transplantation, blood vessels were analyzed with Masson and CD31 staining (**Figure 2D** , left and middle panel). RA FLSs derived EVs showed a higher ratio of vessels/total tissue than the control EVs. Likewise, the proportion of CD31+ ECs in RA FLSs derived EVs was much higher than the control EVs (**Figure 2E** , left and middle panel). Together, these results demonstrate that RA FLSs derived EVs play a key role in facilitating angiogenesis of ECs *in vitro* and the matrigel angiogenesis mice model.

### LPS Stimulated RA FLSs Derived EVs Are More Powerful in Expediting Angiogenesis of ECs

Numerous studies have shown that cell surface expressed Toll-like receptor (TLR) is correlated with RA (34). Moreover, it has been documented that TLR2/4 is highly expressed in synovial tissues and FLSs in RA patients (36, 37). Importantly, LPS from *P. gingivalis* activates TLR2 result in the upregulation of the extracellular matrix protein TSP1 (thrombospondin-1) and IL-33 in monocytes in RA patients (38). Additionally, TLR4 signaling can been induced by LPS in RA FLSs (37). However, whether LPS stimulated RA FLSs derived EVs also aggravate the angiogenesis of ECs and the underlying mechanism is unknown. Hence, we investigated the angiogenesis capacity of LPS stimulated RA FLSs derived EVs using the established systems above. Interestingly, the data showed that LPS stimulated RA FLSs derived EVs significantly promoted tubules generation in matrigel system compared to unstimulated RA FLSs derived EVs (**Figure 2A** , right panel). Both the relative tube length, relative junction count and relative mesh count in LPS stimulated RA FLSs derived EVs were significantly higher than the unstimulated EVs (**Figure 2A**). Furthermore, LPS stimulated RA FLSs derived EVs also facilitated the ECs migration compared to the unstimulated EVs (**Figure 2C** , right panel). Similarly, LPS stimulated RA FLSs derived EVs did not promote ECs proliferation within 24 hours compared to the unstimulated EVs (**Figure S2**). Collectively, our results suggest that the stimulation of RA FLSs with LPS can exacerbate the angiogenesis in ECs.

Additionally, pro-inflammatory cytokines represent a typical inflammatory milieu in RA patients. Therefore, we also compared the angiogenesis effects of pro-inflammatory cytokines IL-1β and TNF-α stimulated EVs respectively. Surprisingly, neither IL-1β nor TNF-α stimulated RA FLSs derived EVs can promote tubules generation in matrigel system compared to Trauma FLSs derived EVs (**Figure S3**). Both the relative tube length, relative junction count and relative mesh count in IL-1β or TNF-α stimulated RA FLSs derived EVs were not significantly increased than Trauma FLSs derived EVs. While LPS stimulated RA FLSs derived EVs can dramatically promote ECs angiogenesis compared to Trauma FLSs derived EVs with enhanced relative tube length, relative junction count and relative mesh count. In short, LPS stimulated EVs but not IL-1β or TNF-α stimulated EVs show enhanced capacities of ECs angiogenesis.

Next, we further investigated the angiogenesis capacity of LPS stimulated RA FLSs derived EVs *in vivo* using the developed matrigel angiogenesis mice model. Consistent with the results *in vitro*, LPS stimulated RA FLSs derived EVs were also efficiency in this model with an elevated ratio of vessels/total tissue than the unstimulated EVs (**Figure 2D** , middle and right panel). Accordingly, the proportion of CD31+ ECs in LPS stimulated RA FLSs derived EVs was dramatically increased than the unstimulated EVs (**Figure 2E** , middle and right panel). Thus, the stimulation of LPS to RA FLSs derived EVs facilitate angiogenesis of ECs in mice model as well.

### RNA Parts in RA FLSs EVs Predominately Stimulate Angiogenesis in ECs

**Figure 3:**
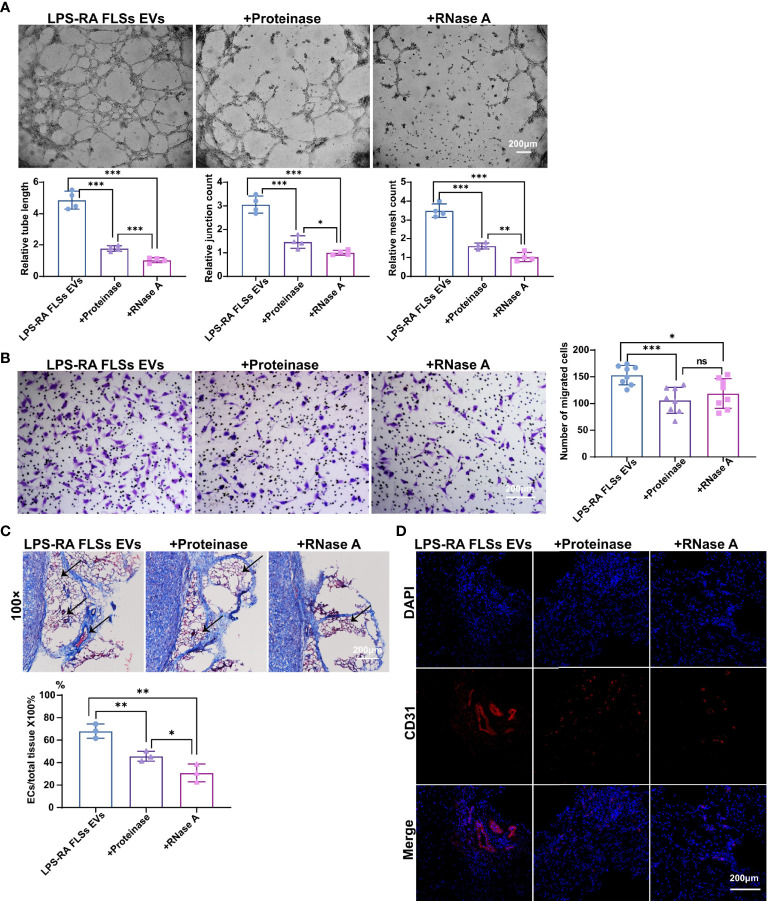
RNAs parts in RA FLSs derived EVs played a pivotal role in promoting ECs angiogenesis. RA FLSs were stimulated with LPS for 3 days. EVs were then obtained using the established procedure. For RNA or protein degradation, EVs were incubated with RNase A or proteinase K. The angiogenesis capacities of EVs were evaluated using the established *in vitro* systems and mice model. **(A)** The tubule formation capacity of RA FLSs derived EVs was determined. Representative photos (50×) were obtained using microscope imaging (panel upper). The results were summarized by calculating the related parameters (panel lower). **(B)** The migration capacity of RA FLSs derived EVs was determined by transwell chamber system. The final concentration of EVs was 50 μg/mL. Typical photos (100×) (panel left), summary data (panel right) are shown. The data indicate the mean ± S.E.M of three independent experiments. **(C, D)** Access of EVs tubule genesis in mice model. **(C)** Masson staining results. Typical photos (100×) (panel upper), summary data (panel lower) are shown. (Red represented endothelial cells and blood vessels, blue represented matrix gel; n=3). **(D)** CD31 fluorescence staining. Typical photos (100×) are shown. (*P < 0.05, **P < 0.01, ***P < 0.001, ns, not significant).

EVs exert their functions predominantly depend on the contents, such as microRNA (miRNA), mRNA, and protein (14, 15). RA FLSs derived EVs are capacity in expediting inflammation and bone erosion *via* membrane-bound TNF-α, IL-6 and IL-8, MMPs contained in the EVs (16–18). Importantly, CD13 and ID1 in RA FLSs derived EVs have been confirmed to facilitate angiogenesis in ECs (19, 21), indicating the proteins involved in RA FLSs derived EVs are able to promote angiogenesis. However, the angiogenesis function of RNAs in RA FLSs derived EVs remain unknown. To investigate the individual role of RNAs or proteins of LPS stimulated RA FLSs derived EVs in angiogenesis, we degraded RNAs or proteins in the EVs using RNase A or proteinase K separately. The efficacies of degraded RNAs or proteins were confirmed with RNA electrophoresis and protein silver staining (**Figures S4A, B**). Thereafter, the angiogenesis capacity was assessed with tubule formation system and migration system individually. The results revealed that both degradation of RNAs or proteins can abolish the tubule formation capacity of LPS stimulated RA FLSs derived EVs, but the effects of RNAs degraded EVs were more significant than the proteins degraded EVs (**Figure 3A** , upper panel). Accordingly, both the relative tube length, relative junction count and relative mesh count in RNAs or proteins depleted EVs were significantly lower than the untreated EVs, notably, the RNAs degraded EVs were much more significant than the proteins degraded EVs (**Figure 3A** , lower panel). However, the promotive capacities of proteins or RNA degraded EVs on ECs migration were indistinguishably decayed dramatically compared to the LPS stimulated RA FLSs derived EVs (**Figure 3B**), reflecting that tubule formation promotive capacities of RNAs and proteins in LPS stimulated RA FLSs derived EVs were embodied in different facets. Additionally, depletion of RNAs or proteins in LPS stimulated RA FLSs derived EVs indistinguishably maintained the proliferation promotive capacity of LPS stimulated RA FLSs derived EVs on ECs (**Figure S4C**), indicating that both RNA and protein parts paly roles in promoting ECs proliferation. Collectively, these data indicate that both RNAs and proteins are involved in the ECs angiogenesis stimulative function of LPS stimulated RA FLSs derived EVs, but RNA parts exert a major role in ECs tubule formation while protein parts mainly in ECs migration.

Deservedly, we verified the results in the matrigel angiogenesis mice model. As expected, the capacity of RNAs depleted EVs were maximizing abrogated in this model with a decreased ratio of vessels/total tissue compared to proteins degraded EVs (**Figure 3C**). Accordingly, the proportion of CD31+ ECs was also dramatically decreased than the proteins depleted EVs (**Figure 3D**). Thus, RNA parts in LPS stimulated RA FLSs derived EVs play key role in stimulating angiogenesis of ECs.

### miR-1972 Is Upregulated in LPS Stimulated RA FLSs Derived EVs

**Figure 4:**
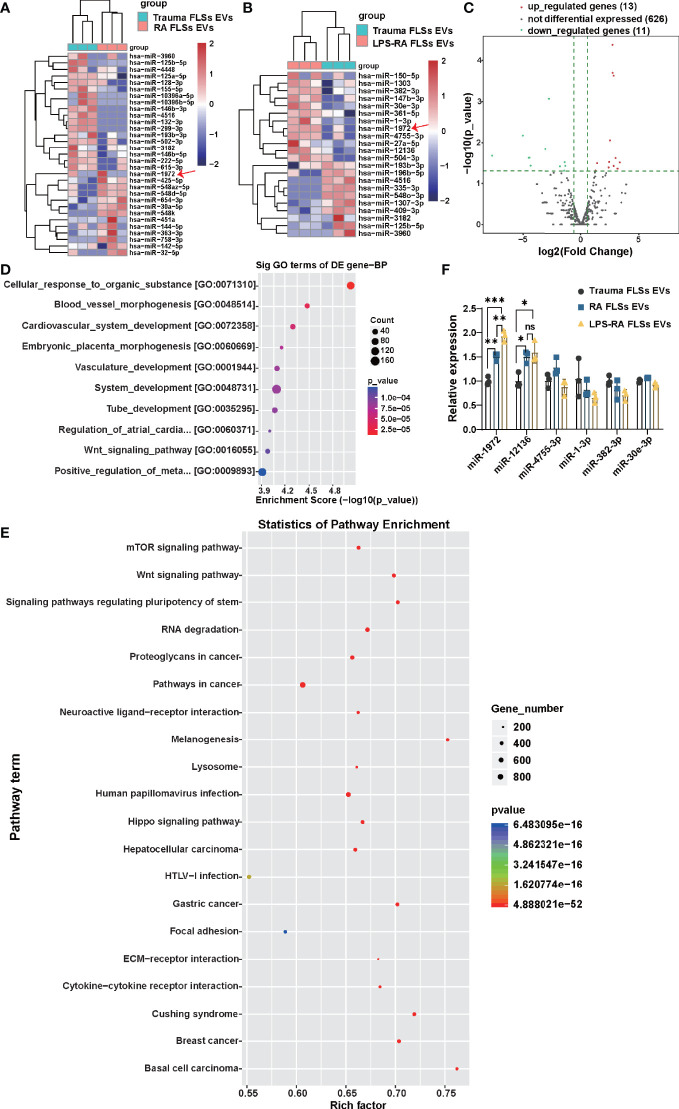
miR-1972 is upregulated in LPS stimulated RA FLSs derived EVs. RNA-seq data of RA FLSs EVs or LPS stimulated RA FLSs EVs versus Trauma FLSs EVs were performed to verify the bioinformatic results. qPCR analysis of RA FLSs versus Trauma FLSs were used to confirm the RNA-seq results. **(A)** The Heat Map was used to show the RNA-seq data of distinguishable miRNA expression profiles in RA FLSs EVs versus Trauma FLSs EVs (fold changes >2 and P <0.05, n=3). **(B)** The Heat Map was used to show the RNA-seq data of distinguishable miRNA expression profiles in LPS stimulated RA FLSs EVs versus Trauma FLSs EVs (fold changes >2 and P <0.05, n=3). **(C)** Expression profile of LPS stimulated RA FLSs EVs versus Trauma FLSs EVs. The results were showed with the volcano plot. **(D, E)** The possible biological functions **(D)** and signaling pathways **(E)** of the differentially expressed miRNAs were analyzed by Gene Set Enrichment Analysis (GSEA) and Gene ontology (GO) analysis. The bubble chart was used to show the partial enrichment results. The color and size of each dot represents the enriched gene number in the GSEA reactome and GO Biological Process. **(F)** The expressions of miRNAs were verified by qPCR in Trauma FLSs EVs and RA FLSs EVs or LPS stimulated RA FLSs EVs. The data indicate the mean ± S.E.M of three independent experiments (n = 3, *P < 0.05, **P < 0.01, ***P < 0.001, ns, not significant).

miRNAs of RA FLSs has been verified to exert a pro-inflammatory role in local joints, modulate the tumor-like behavior of RA FLSs, and result in bone destruction (25–27). However, whether RA FLSs derived miRNAs can regulate the angiogenesis is unclear. Given the conclusion that RNA parts play a key role in facilitating the angiogenesis of ECs, we hypothesis that miRNA in EVs can predominantly regulate angiogenesis of ECs. To accurately identify which miRNAs may function mainly, the miRNA expressions in RA FLSs derived EVs were investigated using miRNA sequencing. In total of 31 miRNAs were significantly changed in the 630 scanned miRNAs in RA FLSs derived EVs, compared to Trauma FLSs derived EVs, with 13 up-regulated miRNAs and 18 down-regulated miRNAs (P<0.05; Fold change≥2) (**Figure 4A**). Furthermore, the miRNA expressions in LPS stimulated RA FLSs derived EVs were investigated. In total of 24 miRNAs were significantly changed in the 650 scanned miRNAs in LPS stimulated RA FLSs derived EVs, compared to Trauma FLSs derived EVs, with 13 up-regulated miRNAs and 11 down-regulated miRNAs (P<0.05; Fold change≥2) (**Figures 4B, C**). Next, we analyzed the possible biological functions of the expression changed miRNAs using GO analysis. Of note, the results showed that expression altered miRNAs may principally correlate with blood vessel morphogenesis, cardiovascular system development, vasculature development, tube development and some other biologic processes (P < 0.05) (**Figure 4D**), which was consistent with the conclusion that RNA parts play key role in stimulating angiogenesis of ECs. Subsequently, we further used KEGG analysis to investigate cellular functions of the expression changed miRNAs in EVs. The results showed that mTOR signaling pathway was the most enriched pathway (**Figure 4E**), which was documented related to angiogenesis in tumor microenvironment (39). It may signify that this pathway also exerts a parallel role in RA. Additionally, we further confirmed the up-regulated miRNAs with qPCR. Notably, miR-1972 and miR-12136 were identified highly expressed in RA FLSs derived EVs compared to Trauma FLSs derived EVs. However, the expression of miR-1972 in LPS stimulated RA FLSs derived EVs was higher than the unstimulated EVs (**Figure 4F**). Thus, by combining with the conclusion that the LPS stimulated RA FLSs can exacerbate the angiogenesis in ECs, miR-1972 is confirmed to mainly stimulate the angiogenesis.

### miR-1972 Directly Stimulates Angiogenesis in ECs

**Figure 5:**
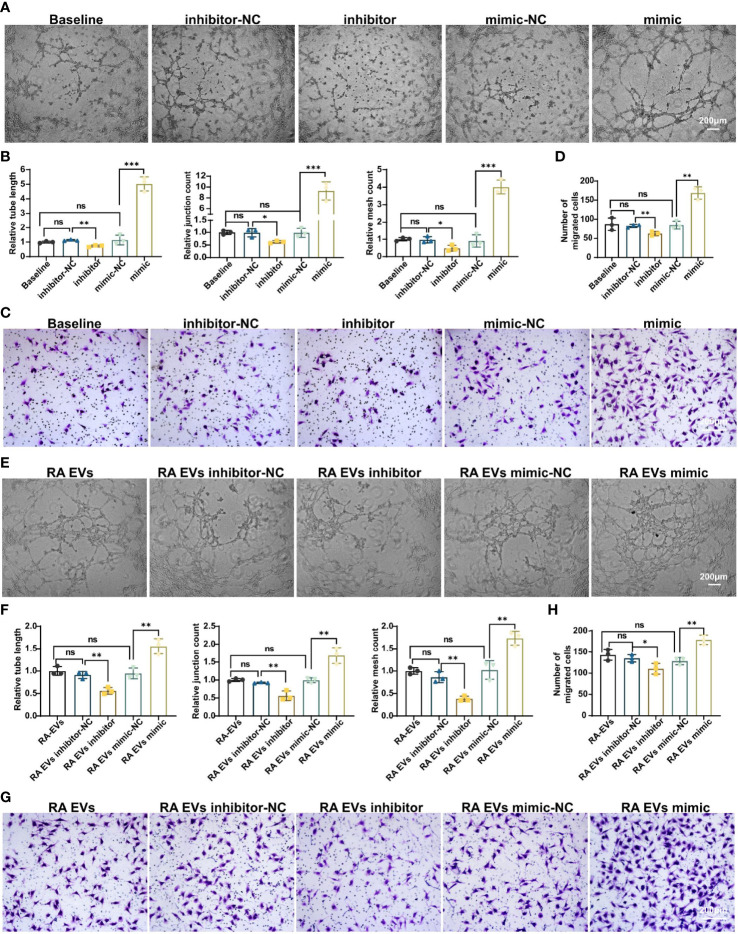
miR-1972 regulates angiogenesis directly in ECs or mediated *via* RA FLSs derived EVs. **(A–D)** ECs in miR-1972 overexpression system or knock down system were prepared for tubule formation or migration detections. The tubule formation capacity of ECs was determined. Representative photos (50×) were obtained using microscope imaging **(A)**. The results were summarized by calculating the related parameters **(B)**. The migration capacity of ECs was determined by transwell chamber system. Typical photos (100×) **(C)**, summary data **(D)** are shown. RA FLSs derived EVs in miR-1972 overexpression system or knock down system were prepared for tubule formation or migration detections. The tubule formation capacity of ECs was determined. Representative photos (50×) were obtained using microscope imaging **(E)**. The results were summarized by calculating the related parameters **(F)**. The migration capacity of ECs was determined by transwell chamber system. Typical photos (100×) **(G)**, summary data **(H)** are shown. The data indicate the mean ± S.E.M of three independent experiments. (n = 3, *P < 0.05, **P < 0.01, ***P < 0.001, ns, not significant).

To investigate whether miR-1972 can function directly in ECs, we established miR-1972 knock down system and overexpression system individually. The efficiencies of the knock down or overexpression were confirmed using qPCR analysis (**Figure S5**). Thereafter, the tubule generation capacities of ECs under the developed systems were performed. Definitely, tubule formation of ECs in miR-1972 knock down system was dramatically decayed compared to the negative control (**Figure 5A** , left and middle panel). Consequently, both the relative tube length, relative junction count and relative mesh count in miR-1972 down regulated ECs were significantly decreased than the control ECs (**Figure 5B**). Reversely, both tubule formation and the relevant parameters in miR-1972 overexpression system were elevated (**Figures 5A, B**). Next, we explored the migration capacities of miR-1972 knock down or overexpressed ECs. The results also showed a reversed phenomenon (**Figures 5C, D**), indicating that miR-1972 can directly promote angiogenesis in ECs.

Furthermore, we explored the function of miR-1972 in RA FLSs derived EVs on angiogenesis of ECs using the miR-1972 knock down or overexpression systems. As expected, tubule formation of RA FLSs derived EVs in miR-1972 knock down system was dramatically decreased compared to the negative control (**Figure 5E** , left and middle panel). Moreover, both the relative tube length, relative junction count and relative mesh count in miR-1972 down regulated EVs were significantly declined than the control ECs (**Figure 5F**). Importantly, the results were reversed when miR-1972 was overexpressed in RA FLSs derived EVs (**Figures 5E, F**). Congruously, expression alterations of miR-1972 in EVs also altered the ECs migration compared to the negative control EVs (**Figures 5G, H**). Together, these data indicated that miR-1972 act as a rheostat in expediting angiogenesis in ECs.

### miR-1972 Exerts Function *via* p53/mTOR Signaling in ECs

**Figure 6:**
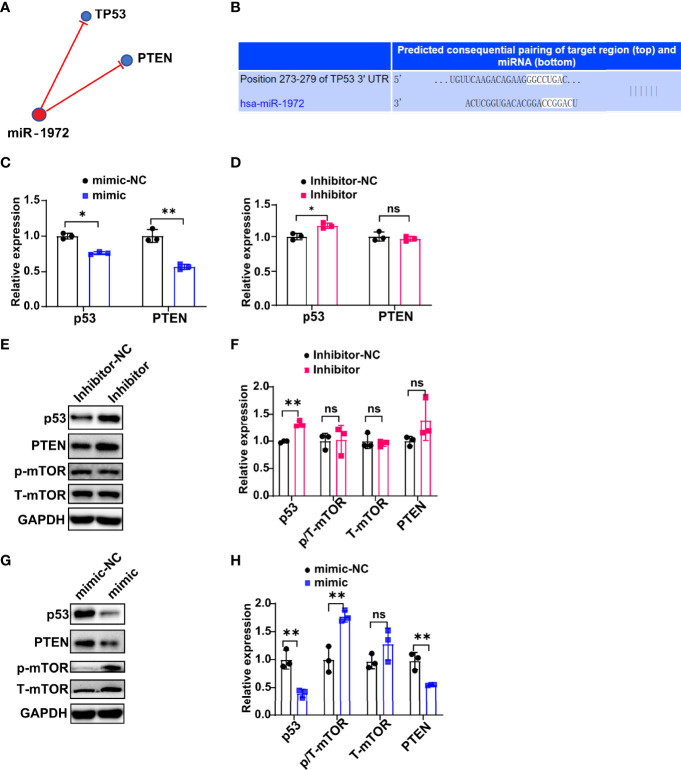
miR-1972 exerts function *via* p53/mTOR signaling in ECs. **(A, B)** Potential related signaling of miR-1972 was predicted using a miRNA targets prediction website (TargetScan, http://www.targetscan.org/) **(A)**. The mRNA that miR-1972 targeted complementally was blasted using TargetScan **(B)**. **(C, D)** The expressions of ECs p53 and PTEN in miR-1972 overexpression system **(C)** or knock down system **(D)** were verified by qPCR. **(E, F)** The expression changes of ECs p53, PTEN and mTOR in miR-1972 knock down system were detected using western blotting. Typical photos **(E)**, summary data **(F)** are shown. **(G, H)** The expression changes of ECs p53, PTEN and mTOR in miR-1972 overexpression system were detected using western blotting. Typical photos **(G)**, summary data **(H)** are shown. The data indicate the mean ± S.E.M of three independent experiments. (n = 3, *P < 0.05, **P < 0.01, ns, not significant).

To investigate the underlying mechanism that how miR-1972 exerts function, we predicted the potential related signaling of miR-1972 using a miRNA targets prediction website (TargetScan, http://www.targetscan.org/). The results showed that miR-1972 may negatively regulate TP53 (also known as p53) signaling and PTEN signaling (**Figure 6A**). Specifically, miR-1972 was predicted bind to TP53 mRNA complementally (**Figure 6B**). Next, we used the overexpression or knock down systems to verify if miR-1972 can modulate p53 signaling and PTEN signaling in ECs. The qPCR results revealed that both the mRNA levels of p53 and PTEN were decreased in the overexpression system (**Figure 6C**), while only p53 mRNA was reversed in the knock down system (**Figure 6D**), indicating that miR-1972 may function mainly *via* p53 signaling. Importantly, it has been reported that both p53 and PTEN pathways are involved in regulating angiogenesis. Therefore, we speculated that miR-1972 might expedite angiogenesis through p53 or PTEN pathways. To explore the which pathways are more correlated to miR-1972 in ECs, we detected the changes of p53, PTEN, mTOR and phosphorylated mTOR in miR-1972 knock down or overexpression systems. The results showed that p53 but not PTEN level was significantly enhanced in miR-1972 knock down system (**Figures 6E, F**), which confirmed that miR-1972 function *via* p53 pathway. However, both p53 and PTEN levels were significantly decreased in miR-9172 overexpressed ECs (P<0.01) (**Figures 6G, H**). Inversely, phosphorylated mTOR was specifically elevated without changing total mTOR level (**Figures 6G, H**), implying that miR-1972 may enhance mTOR phosphorylation by negatively regulating p53 or PTEN levels. Collectively, these data indicate that miR-1972 promote angiogenesis specifically through negatively regulating p53 and then elevating mTOR phosphorylation.

## Discussion

Rheumatoid arthritis is a chronic autoimmune disease characterized by inflammation, polyarticular synovitis and bone loss (1). Pannus formation is one of the driving pathologic processes which can lead to the development of joint erosion in RA (2). The possibility of mitigating the generation of pannus by interfering with angiogenesis has been verified efficacy in animal models of arthritis. Thus, it is a potential target by blocking angiogenesis in the treatment of RA (8). Indeed, vascular targeted candidates have been developed in pre-clinic study (9). However, the current candidate development cannot meet the urgent demand of RA patients. Meanwhile, the underlying mechanisms of pannus generation in RA are not completely uncovered. Therefore, it is important to clarify the potential mechanism that contribute to RA pannus formation.

RA FLSs is a crucial determinant of RA pathogenesis that undergo abnormal activation by pro-inflammatory cytokines, interact with neutrophils to accelerate pathogenic adaptive immunity, and contribute to joint damage (40–42). Accumulating evidence showed activated RA FLSs possess lots of biological characteristics, such as hyperproliferation, migration and tissue invasion which are similar to tumor cells (27). The tumor-like biologic characteristics of RA FLSs are further lead to pannus formation, recruiting inflammatory cells infiltrate into local joints and resulting in cartilage destruction and bone erosion. Therefore, it is crucial to elucidate the underlying mechanism that how RA pannus is generated and form network with other cells in joint milieu. Here, consistent with others, we also confirmed that RA FLSs can promote ECs angiogenesis.

Pathologically, RA FLSs have been documented to regulate ECs angiogenesis by producing cytokines, growth factors, chemokines, and adhesion molecules. Also, RA FLSs derived EVs possess multifunction in expediting the pathology of RA by a T cell-to-RA FLSs interaction feedback loop. Here, we showed that RA FLSs derived EVs accelerate ECs angiogenesis by promoting ECs tube formation, migration and proliferation. Importantly, the EVs also function well in matrigel angiogenesis mice model with an increased tubule generation and CD31 expression in ECs. Moreover, LPS can activate RA FLSs result in the their pro-inflammatory phenotype (4, 38). In this study, interestingly, we clarified LPS stimulated RA FLSs also aggravate ECs angiogenesis, underlining the key role of RA FLSs in stimulating pannus formation in RA.

RA FLSs exert function through secreting multiple factors. miRNAs have been verified involved in regulating RA FLSs functions. miR-221-3p expedites tumor-like behavior of RA FLSs *via* uPAR pathway (27). miRNA-146a-5p can suppress pannus formation in CIA mice (2). miR-1972 has been identified to facilitate cell viability, invasion and metastasis *via* a ceRNA network in osteosarcoma (31, 32). Furthermore, a APCDD1L-AS1-miR-1322/miR-1972/miR-324-3p-SIRT5 axis has been investigated to facilitate icotinib-resistance by suppressing autophagic degradation of EGFR in lung adenocarcinoma. Importantly, miR-1972 has been verified to decrease the proliferation, and/or migration as well as tube formation of ECs in preeclampsia (33). Here, consistent with others, we show that miR-1972 promote angiogenesis through negatively regulating p53 and then elevating mTOR phosphorylation. Target on miRNAs may exert a therapeutic role in the treatment of RA. Local injection of liposomes with miR-17-5P mimic in arthritis mice joints can significantly alleviate inflammation and articular damage by directly targeting STAT3 and JAK/STAT pathways (43). Therefore, target on miR-1972 may also have a therapeutic effect in RA patients.

In terms of mechanism, lots of pathways have been documented function the tumor-like behavior of RA FLSs. SHH-JNK signaling has been verified to suppresses the aggressiveness of RA FLSs (44). uPAR-PI3K/Akt pathway promotes tumor-like behavior of RA FLSs (45). Also, numerous studies have revealed that the activation of PI3K/Akt/mTOR pathway is involved in ECs angiogenesis (46, 47), and p53 can suppress this pathway (48). Therefore, p53 is considered to be a key checkpoint of angiogenesis (49). In our study, consistent with others, we revealed that miR-1972 negatively regulates p53 level and enhances mTOR phosphorylation. In ECs angiogenesis, activated PI3K/Akt/mTOR pathway can promote the survival and proliferation of ECs (50). Here, we further confirmed that p53/mTOR signaling also involved in angiogenesis of ECs.

In summary, our data indicate that RA FLSs derived EVs promote ECs angiogenesis by enhancing migration and tube formation of ECs *in vitro* and a matrigel angiogenesis mice model. In terms of the mechanisms, miR-1972 is identified to facilitate ECs angiogenesis. The blockage of miR-1972 significantly abrogated the angiogenesis stimulative ability of RA FLSs derived EVs in ECs, while the overexpression of miR-1972 reversed the effect in ECs. Collectively, RA FLSs derived EVs can promote ECs angiogenesis *via* miR-1972 targeted p53/mTOR signaling (**Graphical Abstract**), targeting on RA FLSs derived EVs or miR-1972 provides a promising strategy for the treatment of patients with RA.

## Material and Methods

### Patients

Synovial tissues were collected from active RA patients undergoing synovectomy of the knee joint or total knee replacement surgery at the Third Affiliated Hospital of Sun Yat-sen University. RA patients were diagnosed according to the 2010 American College of Rheumatology/European League Against Rheumatism (ACR/EULAR) classification criteria. For negative control, synovial tissues were taken from nine patients who underwent arthroscopic surgery for severe joint trauma without other joint abnormalities or systemic diseases. The protocols were approved by the ethics committee of the Third Affiliated Hospital at Sun Yat-sen University, and all subjects provided written informed consent in accordance with the Declaration of Helsinki. For experiments, FLSs from each donor were employed in one experiment. At least three donors derived FLSs were used in all experiments.

### Isolation of FLSs

FLSs were prepared as we previously reported (45). Briefly, synovial tissues were dissected and rinsed 2–3 times with phosphate-buffered solution (PBS), repeatedly shredded into ~ 1 mm^3^ pieces, incubated in flasks. The flasks containing an appropriate amount of DMEM (Gibco) culture medium supplemented with 10% fetal bovine serum and were placed in 37°C, 5% CO_2_ thermostatic incubator. The cells were considered as type B fibroblast-like synovial cells when the typical spindle-shaped, fibroblast-like appearance was present and surface marker CD90 was positive. FLSs from passages 3 to 6 were used for the following experiments.

### Cell Line

Human umbilical vein endothelial cell line (EA. hy926) (51), was purchased from ATCC. Cells were cultured in High Glucose Dulbecco’s Modified Eagle’s Medium (DMEM; Gibco) containing 10% fetal bovine serum (FBS) and supplemented with 1% Penicillin and Streptomycin under 5% CO_2_ at 37°C in a humidified atmosphere.

### Identification of FLSs

The obtained FLSs were identified using the surface markers. The following fluorescence conjugated human mABs were used for flow cytometry analysis from BioLegend (San Diego, CA): APC-CD90 (5E10), PerCP/Cy5.5-CD11b (ICRF44), PE-CD29 (TS2116), APC-CD68 (Y1182A), FITC-CD44 (IM7). Cell subsets were stained with human antibodies and isotype control indicated above. Samples were examined using FACS Calibur flow cytometer and analyzed using Cell Quest Software (Becton, Dickinson). Final histogram figures were prepared with FlowJo Software (Tree Star, Ashland, OR).

### Isolation of Extracellular Vesicles (EVs)

To obtain a dependable EVs, the any existed EVs in FBS were removed by centrifugation (100,000 g, at 4°C) for 24 hours. The upper serum was carefully collected and filtrated with a 0.22 μm needle filter for the next step. For the preparation of RA or Trauma FLSs derived EVs, the FLSs were incubated with EVs depleted FBS for 72 h. The supernatants were centrifuged at 300 g for 10 min, and another 3000 g for 20 min at 4°C to remove any cells and debris. Next, the supernatants were filtrated with 0.8 µm filter and then a Millipore 100 kD ultrafiltration tube to concentrate the supernatants. The EVs were extracted using QIAGEN’s commercialized exoEasy Maxi Kit (Cat.No.76064). The isolated EVs can be directly used for identification, while for the functional experiments, the EVs was eluted twice with PBS in Millipore 100 kD ultrafiltration tube.

### Identification of EVs

Morphology of EVs were examined with Transmission Electron Microscope (JEM-1200EX, Japan). Briefly, 10 μL of EVs suspension was loaded onto a carbon-coated copper grid, standing at room temperature. EVs was rinsed with PBS incubated with 3% phosphotungstic acid at room temperature for 5 min. After fixed at least 5 min, the grids were visualized and photographed with transmission electron microscope at 80 kV.

The size range of extracellular vesicles was analyzed with Nanoparticle Tracking Analysis (NTA, NanoSight NS300, Malvern, UK) (52). The particles can be automatically tracked and sized according to Brownian motion and diffusion coefficient. EVs were suspended in 1 mL PBS, while PBS alone was used as blank control. The temperature of NTA was 23 ± 0.5°C, the measurement time was 60 S (25 frames per second). Each sample was counted three times. The protein concentration in EVs was detected with BCA method.

### Western Blot

Proteins were prepared as we previously documented (27). Total proteins were extracted from cells with RIPA lysis buffer mixed with phenylmethyl-sulfonyl fluoride (PMSF) and phosphatase inhibitor cocktail I (MedChemExpress, China). An equal amount of protein was separated on 10% or 12% SDS-PAGE gels based upon the molecular weight of the target protein and transferred to polyvinylidene difluoride (PVDF) membranes (Millipore, USA). Membranes were blocked with 5% bovine serum albumin (BSA) at room temperature for 1 h and then incubated overnight at 4°C with primary antibodies (anti-CD9 antibody, abcam, USA, #ab92726; anti-CD63 antibody, abcam, USA, #ab213090; anti-CD81 antibody, abcam, USA, #ab109201; anti-TSG101 antibody, abcam, USA, #ab83; anti-p53 antibody, abcam, USA, #ab179477; anti-PTEN antibody, Cell Signaling Technology, USA, #9559S; anti-mTOR antibody, Cell Signaling Technology, USA, #2983T; anti-phospho mTOR antibody, Cell Signaling Technology, USA, #5536T; anti-GAPDH antibody, SIGMA, USA, #G9545). Subsequently, proteins were detected by an enhanced chemiluminescence (ECL) system reagent (KeyGEN BioTECH, China) after incubated with horseradish peroxidase-conjugated secondary antibodies for 1 h at room temperature. Protein expression was calculated with Image J software and normalized to GAPDH expression.

### Localization of EVs in ECs

For fluorescence staining of EVs, 20 μg RA FLSs or Trauma FLSs derived EVs were incubated with a fluorescent dye PKH67 for 5 min at room temperature, according to the instruction of MINI67-1KT(SIGMA). EVs were then washed twice with PBS. In total of 2.5×10^4^ ECs were seeded on the cell slides in 24-well plate for adhesion. PKH67 labeled EVs were incubated with ECs for 24 h. Endothelial cell nuclei were stained with DAPI. Then, the images were viewed and captured using fluorescence microscope.

### Tubule Formation Assay

To explore the angiogenesis capacity of EVs, tubule formation assay was employed as previously reported (53). Briefly, 60 μl of matrigel matrix (Corning, USA, #354262) was transferred to a prechilled 96-well plate, incubated at 4°C for 5 min, then placed at room temperature for 10 minutes, and finally at 37°C for 30-40 min. ECs were mixed with EVs, and the mixtures were added into the matrix slowly and evenly, the final number ECs were 3×10^4^/well. Tubule formation was observed, and images were collected using inverted microscope (ZEISS, Germany) post 8 hours of culture. The number of meshes (NB meshes), junctions (NB Junctions) and total length of tube (Total length) were calculated with the angiogenesis plug-in of Image J software.

### Cell Migration Assay

Cell migration experiments were carried out in a 24-well transwell chamber (Corning, Cambridge, with an aperture of 8 μm, MA, USA) system (27). In total of 5 × 10^3^ cells were suspended in 2% fetal bovine serum and inoculated in the upper chamber. Then, 600 μl of medium containing 5% fetal bovine serum was added as a chemical inducer in the lower chamber. After incubation at 37°C in 5% CO_2_ for 24 h, non-migratory cells were removed from the upper surface of the filter with cotton swabs. Cells that had migrated through the membrane were fixed in 4% paraformaldehyde (Boster, China) for 20 minutes, stained with crystal violet for another 20 minutes, and counted under a microscope. The number of migrated cells was calculated as the average number of cells passing through the membrane in five randomly selected regions.

### CCK-8 Assay

For ECs proliferation experiment, CCK-8 assay was used as previously described with slight modifications (54). Specifically, ECs were inoculated in 96-well plates at different time points with different densities (6×10^3^/well for 24 h, 5×10^3^/well for 48 h, and 4×10^3^/well for 72 h). After the cells adhered, FLSs derived EVs were added into the wells. Cells were incubated with CCK-8 (ESscience, China, #ES7011) at 37°C for 1 h protect from light. The absorbance was detected at 450 nm using BioTek Synergy H1MF(USA). The proliferation rate was expressed as the *OD* values. All the experiments were undertaken three times and showed as mean ± S.E.M.

### Matrigel Angiogenesis Mice Model

To determine angiogenic potential of EVs *in vivo*, matrigel mixed with ECs and EVs was injected subcutaneously into NOD SCID mice (55). For the experiment setup, NOD SCID mice were assigned to three groups: Trauma FLSs EVs (500 μl matrigel and 1×10^6^ ECs mixture), RA FLSs EVs (500 μl matrigel and 1×10^6^ ECs mixture), and LPS stimulated RA FLSs EVs (500 μl matrigel and 1×10^6^ ECs mixture). The final concentration of EVs was 150 μg/ml. Matrigel mixtures were subcutaneously injected into the lower dorsal region of SCID mice. Two weeks post the transplantation, the matrigel plugs were removed, fixed in 4% paraformaldehyde for 24 h. Then, the specimens were made to frozen slides for masson staining (Servicebio, China, #G1006) and CD31(Servicebio, China, #GB11063-2) immunofluorescence staining according to the instruction. Images were taken under a positive microscope (Leica, Germany), and the proportions of ECs and blood vessels were analyzed using image J.

### Elimination of RNAs or Proteins in EVs

To remove RNAs or proteins of EVs, the EVs were put in liquid nitrogen for 1-2 minutes, and then dissolved in 37°C water bath for 2-3 minutes. EVs were underwent five freeze-thaw cycles as described previously (56). Thereafter, the EVs were treated with RNase A (Takara, 100 μg/mL, 37°C, 30 min) to degrade RNAs in EVs, followed by incubating with RNase A inhibitor (Takara, 2,000 units/mL, 37°C, 30 min) to inactivate RNase A. To degrade proteins, EVs were treated with proteinase K (Sigma, 25 μg/mL, 37°C, 30 min), followed by heating at 95°C for 5min to inactivate proteinase K.

### Agarose Gel Electrophoresis of RNAs

Agarose gel (1.0%) was prepared with 1×TAE. RNAs of EVs were extracted and mixed with Loading Buffer. In the horizontal electrophoresis tank, the samples were electrophoresed at 80 V for 10-20 minutes. Images were taken with gel imager (Syngene, UK) to verify whether the RNAs was degraded.

### Silver Staining of Proteins

To verify the degradation efficiency of EVs proteins, the proteins were electrophoresed on the 10% SDS-PAGE gel. Then the gel was silver stained with protein silver staining kit (Pierce™ Silver Stain for Mass Spectrometry, Thermo Fisher). Photos were taken to verify whether the EVs proteins were degraded.

### EVs microRNA-Sequencing

Total RNA of EVs was extracted with trizol LS (Invitrogen, USA), and quantified with Nanodrop. Agarose electrophoresis was used for quality inspection, and library was constructed after passing the quality inspection. Then, Agilent 2100 Bioanalyzer was used to determine the quality of the library. RNA was denatured with 0.1 M NaOH and sequenced on Illumina NextSeq500. The sequencing results were compared well with the reference genome. The up-regulation or down-regulation of miRNA was defined according to the threshold of P<0.05 and fold change>2. The cluster diagram, scatter diagram and volcano diagram were drawn. The differential target genes of microRNAs were enriched by GO and analyzed by KEGG pathway.

### GSEA and GO Analysis

Gene Set Enrichment Analysis (GSEA) and Gene Ontology (GO) analysis were used to identify characteristic biological processes in which miRNAs may participate. Gene Set Enrichment Analysis (GSEA) is a computational method that can determine whether a predefined set of genes show a statistically significant and consistent difference between two biological states. GO analysis covers three domains: Biological Process, Cellular Component and Molecular Function.

### Quantitative Real-Time Reverse Transcription PCR

Total RNA from cells was extracted using TRIzol RNA Reagent (Invitrogen, Carlsbad, CA, USA) in accordance with the manufacturer’ s instructions, and total RNA from EVs was extracted using TRIzol LS Reagent (Invitrogen, Carlsbad, CA, USA). The main steps were shown in **Supplementary Table 1**. NanoDrop ND-2000 (Thermo Fisher Scientific, Waltham, MA) was used to measure the quantity and quality of RNA samples. To determine the relative expression and transfection efficiency of miR-1972, cDNA was synthesized by equal amounts of RNA from different samples using Mir-X miRNA First-Strand Synthesis Kit (Takara, Mountain View, CA, USA; #638313) and detected using TB GreenTM Premix Ex Taq™II PCR (Takara Bio-technology; #RR820A). All qPCR reactions were performed on ABI 7500 real-time PCR amplification equipment (Thermo Fisher, QuantStudio 5, USA). The PCR primers were listed in **Supplementary Table 2**. The relative expression of target genes was normalized to the internal reference genes U6 and was calculated using the 2^-△△Ct^ method.

### Transfection of miR-1972 Mimic and Inhibitor

miR-1972 was down expressed or overexpressed using LIPO3000 and microRNA inhibitor or mimic systems in ECs (in 6-well plate, 2 mL system) and RA FLSs (in 100 mm dish, 6 mL system). Briefly, cells were seeded in 6-well plate (ECs) or 100 mm dish (FLSs) 18 h before transfection, and the cells reached 60% confluence. The specific transfection procedures were as follows (at room temperature): (1) Solution A was prepared: Opti-MEM (125 μL/mL) was mixed with LiPO3000 and incubated for 5 min. (2) Solution B was prepared: Opti-MEM (125 μL/mL) and miR-1972 inhibitor/mimic (and corresponding control) and incubated for 5 min. (3) The above A plus B solution was thoroughly mixed, incubated for 15-20 min, and cultured with DMEM containing 10% fetal bovine serum after 8 h incubation. Subsequent experiments were performed 3 days after infection.

### Statistics

The data presented were derived from at least 3 independent experiments. Statistical analysis was performed by SPSS version 25.0 software (SPSS Inc., Chicago, IL, USA). Experimental data are presented as the mean ± Standard Error of Mean (S.E.M). Student’s t-test was used for data comparison between two groups, and differences were considered statistically significant when P-values were less than 0.05.

## Data Availability Statement

The datasets presented in this study can be found in online repositories. The names of the repository/repositories and accession number(s) can be found below: https://www.ncbi.nlm.nih.gov/geo/query/acc.cgi?acc=GSE185672.

## Ethics Statement

The research was approved by the ethics committee of the Third Affiliated Hospital at the Sun Yat-sen University and all subjects were given the written informed consent in accordance with the Declaration of Helsinki.

## Author Contributions

YLu, YFP, and YXC designed the experiments. YXC, JLD, XRL, MLW, and YLiu performed the experiments. XQL, WZW, and ZYH collected synovial tissues. YLiu, JRC, YC, XQL, XYS, and XB analyzed these data. YXC wrote the manuscript. YLu and YFP edited the manuscript. All authors contributed to the article and approved the submitted version.

## Funding

This work was supported by grants provided from the National Natural Science Foundation of China (No.81771750), the National Natural Science Foundation of China (82101899 to YL) and China Postdoctoral Science Foundation Grant (2021M693658 to YL).

## Conflict of Interest

The authors declare that the research was conducted in the absence of any commercial or financial relationships that could be construed as a potential conflict of interest.

## Publisher’s Note

All claims expressed in this article are solely those of the authors and do not necessarily represent those of their affiliated organizations, or those of the publisher, the editors and the reviewers. Any product that may be evaluated in this article, or claim that may be made by its manufacturer, is not guaranteed or endorsed by the publisher.

## Supplementary Material

Supplementary Figure S1 Morphology and surface marker of the obtained FLSs. RA FLSs or Trauma FLSs were isolated according to the previous method. Morphology of the FLSs were imaged using microscope **(A, B)**. The phenotype of FLSs were detected using flow cytometry. Typical histogram figures of RA FLSs and Trauma FLSs are shown **(C)**.

Supplementary Figure S2 RA FLSs derived EVs do not affect ECs proliferation. RA FLSs or Trauma FLSs derived EVs were obtained using the established procedure and cultured with ECs in proliferation system. For LPS stimulation, RA FLSs were cultured in DMEM with LPS (1 μg/mL) for 3 days. *OD* values were detected at 24, 48 and 72 hours, respectively. The summarized results are shown. The data indicate the mean ± S.E.M of three independent experiments. (*P < 0.05, **P < 0.01, ns, not significant).

Supplementary Figure S3 Different pro-inflammatory cytokines stimulated EVs effect ECs angiogenesis. RA FLSs or Trauma FLSs derived EVs were obtained using the established procedure and cultured with ECs in tube formation system. For LPS stimulation, RA FLSs were cultured in DMEM with LPS (1 μg/mL) for 3 days. For pro-inflammatory cytokines stimulation, RA FLSs were cultured in DMEM with IL-1β (1 ng/mL, R&D, 201-LB-010/CF) or TNF-α (10 ng/mL, PeproTech, 300-01A) for 3 days. The summarized results are shown. The data indicate the mean ± S.E.M of three independent experiments. (*P < 0.05, **P < 0.01, ns, not significant).

Supplementary Figure S4 Efficiencies of RNase A or proteinase K in removing RNA or protein of EVs, and effects on ECs proliferation. RA FLSs were stimulated with LPS for 3 days. EVs were then obtained using the established procedure. For RNA or protein degradation, EVs were incubated with RNase A or proteinase K. For ECs proliferation assay, EVs were cultured with ECs in proliferation system. *OD* values were detected at day1. **(A)** RNA levels of RNase A disposed EVs are shown by agarose gel electrophoresis image. **(B)** Protein levels of proteinase K disposed EVs are shown by silver staining image. **(C)** The summarized ECs proliferation results are shown. The data indicate the mean ± S.E.M of three independent experiments. (*P < 0.05, **P < 0.01, ns, not significant).

Supplementary Figure S5 The efficiencies of miR-1972 knock down or overexpression in RA FLSs or ECs. RA FLSs or ECs were cultured in 6-well plate. miR-1972 knock down or overexpression of RA FLSs or ECs were performed in the established systems. qPCR analysis of RA FLSs **(A)** or ECs **(B)** versus control were used to confirm the efficiencies of miR-1972 knock down or overexpression. The data indicate the mean ± S.E.M of three independent experiments (n = 3, *P < 0.05, **P < 0.01, ***P < 0.001, ns, not significant).
