# Supplementary material for: RA Fibroblast-Like Synoviocytes Derived Extracellular Vesicles Promote Angiogenesis by miRNA-1972 Targeting p53/mTOR Signaling in Vascular Endotheliocyte
Source: Front Immunol. 2022 Mar 8;13:793855. doi: 10.3389/fimmu.2022.793855 (PMC8957937; doi:10.3389/fimmu.2022.793855)
Supplement: Supplementary file 6 [file Table_1.docx]

**Supplementary Information**

**Supplementary table 1**

| Steps | Addition Quantity | Placement Temperature, Time | Centrifuge(4°C), Time |
| --- | --- | --- | --- |
| 1. Trizol (cells); Trizol LS(EVs) | Trizol; Trizol LS: EVs 3:1 | 25 °C, 5 min | / |
| 2. Chloroform | 1/5 Trizol or Trizol LS mixed system | 25°C, 10 min | 12000 g, 15 min |
| 3. Isopropanol | 1.5 times of the supernatant | -80 °C, 2 hours | 25000 g, 20 min |
| 4. 75%ethyl alcohol | 1.2 mL | / | 25000 g, 15 min |
| 5. Dry RNA | / | 25 °C, 5 min | / |
| 6. Solubilize RNA | 20-30 μl DEPC water | / | / |

**Supplementary table 2**

| gene | sequence |
| --- | --- |
| U6 | F：5’-GGAACGTATCAGAGAAGATTAGC-3’  R：5’-TGGAACGCTTCACGAATTTGCG-3’ |
| hsa-miR-1972 | F：5’-GTCAGGCCAGGCACA-3’  R：5’-CCAG TTTTTTTTTTTTTTTGAGCCA-3’ |
| has-miR-12136 | F：5’-CGCAGGAAAAAGTCATGGA-3’  R：5’-CCAGTTTTTTTTTTTTTTTGGCCT-3’ |
| hsa-miR-4755-3p | F：5’-AGCCAGGCTCTGAAGGGAAAGT-3’  R：mRQ 3’ Primer universal downstream (Mir-X miRNA First-Strand Synthesis Kit; Cat. No. 638313) |
| hsa-miR-382-3p | F：5’-CAGAATCATTCACGGACAACA-3’  R：mRQ 3’ Primer universal downstream |
| hsa-miR-30e-3p | F：5’-GCAGCTTTCAGTCGGATGT-3’  R：mRQ 3’ Primer universal downstream |
| hsa-miR-1-3p | F：5’-CGCGTGGAATGTAAAGAAGTATGTAT-3’  R：mRQ 3’ Primer universal downstream |
